# Know your dose: *RADDOSE*

**DOI:** 10.1107/S0907444910006724

**Published:** 2010-03-24

**Authors:** Karthik S. Paithankar, Elspeth F. Garman

**Affiliations:** a Department of Biochemistry, Laboratory of Molecular Biophysics, University of Oxford, South Parks Road, Oxford OX1 3QU, England

**Keywords:** radiation damage, absorbed dose, Compton scattering, X-ray fluorescent escape, Compton electrons, macromolecular crystallography, *RADDOSE*

## Abstract

The program *RADDOSE* is widely used to compute the dose absorbed by a macromolecular crystal during an X-ray diffraction experiment. A number of factors affect the absorbed dose, including the incident X-ray flux density, the photon energy and the composition of the macromolecule and of the buffer in the crystal. An experimental dose limit for macromolecular crystallography (MX) of 30 MGy at 100 K has been reported, beyond which the biological information obtained may be compromised. Thus, for the planning of an optimized diffraction experiment the estimation of dose has become an additional tool. A number of approximations were made in the original version of *RADDOSE*. Recently, the code has been modified in order to take into account fluorescent X-­ray escape from the crystal (version 2) and the inclusion of incoherent (Compton) scattering into the dose calculation is now reported (version 3). The Compton cross-section, although negligible at the energies currently commonly used in MX, should be considered in dose calculations for incident energies above 20 keV. Calculations using version 3 of *RADDOSE* reinforce previous studies that predict a reduction in the absorbed dose when data are collected at higher energies compared with data collected at 12.4 keV. Hence, a longer irradiation lifetime for the sample can be achieved at these higher energies but this is at the cost of lower diffraction intensities. The parameter ‘diffraction-dose efficiency’, which is the diffracted intensity per absorbed dose, is revisited in an attempt to investigate the benefits and pitfalls of data collection using higher and lower energy radiation, particularly for thin crystals.

## Abstract 2

The program *RADDOSE* computes the dose absorbed by a macromolecular crystal and here a guide is provided to help to ensure the proper use of the program. In the new version (v.3) described here, modifications to include the energy deposited owing to Compton scattering have been made.

## 1. Introduction

Synchrotron radiation has become a ubiquitous tool in macromolecular structure determination (Mitchell *et al.*, 1999 ▶; Artz *et al.*, 2005 ▶; Blundell, 2005 ▶). Undulator sources capable of producing high-brilliance beams have led to radiation-damage problems in MX, even at cryotemperatures (Garman & Nave, 2002 ▶; Nave & Garman, 2005 ▶; Ravelli & Garman, 2006 ▶). The damage to a sample at 100 K is proportional to the absorbed dose (deposited energy per unit mass) and is manifest as an overall decrease in diffracted intensity and resolution and increases in unit-cell volume and atomic *B* factor, as well as the reduction of metal centres in metalloenzymes. In addition, specific structural damage to disulfide bonds, to active-site residues and/or to carboxyl groups as well as to other residues (Burmeister, 2000 ▶; Ravelli & McSweeney, 2000 ▶; Weik *et al.*, 2000 ▶) may limit the quantity and quality of biological information that can be obtained.

The dose that can be tolerated by a macromolecular crystal before it loses half of its diffraction intensity was predicted by Henderson (1990 ▶) to be 20 MGy, by analogy with the lifetime exhibited for biological samples in electron microscopy. Owen *et al.* (2006 ▶) experimentally measured a dose limit of 43 MGy (dose to half diffraction intensity) for MX, but recommended a maximum dose of 30 MGy in order that the biological information obtained was not compromised. This dose, the so-called ‘experimental dose limit’, corresponds to a reduction of the average total diffraction intensity to 0.7 of its original value. The crystal may not last up to this limit owing to chemical factors (*e.g.* particularly if there are susceptible residues at crystal contacts), but it is not expected to outlive it. The nature of the specimen under study contributes to the amount of energy absorbed, *i.e.* for the same incident flux density (photons s^−1^ mm^−2^) a sample derivatized with heavy-metal atoms absorbs more energy in a given time (leading to a greater absorbed dose) compared with its native counterpart.

Three major processes occur when an X-ray photon interacts with a macromolecular crystal: photoelectric absorption, inelastic scattering and elastic scattering. During photoelectric absorption all the energy of the incident photon is absorbed by the atom and an electron is ejected. After the absorption event this atom will thus have lost an electron and have an inner shell electron vacancy, which is filled from an outer shell. The excess energy may be released in the form of an Auger electron or by X-ray fluorescence, depending on the incident photon energy, the thickness of the sample and the fluorescence yield of the atom. Secondly, the photon may undergo inelastic Compton scattering, in which some of the energy of the incident photon is transferred to an electron in an atom which recoils and a photon with lower energy is emitted incoherently. The energy of the recoil electron is absorbed in the crystal and contributes to the absorbed dose. Finally, in the case of elastic (coherent or Rayleigh) scattering, the photon is elastically scattered and no energy is deposited in the sample. This process results in the diffraction pattern.

**Figure 1:**
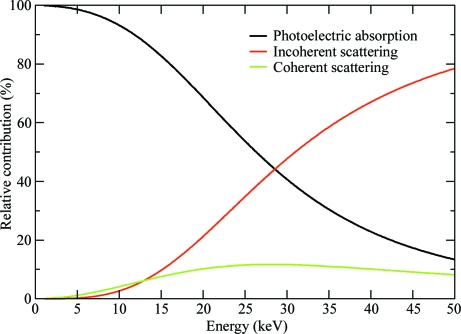
Relative contributions (%) to the total X-ray interaction cross-section for a chicken egg-white lysozyme crystal of dimensions 0.1 × 0.1 × 0.1 mm for a beam of equal size.

The program *RADDOSE* (Murray *et al.*, 2004 ▶; Paithankar *et al.*, 2009 ▶) is widely used to compute the dose (in grays, where 1 Gy = 1 J kg^−1^) absorbed by a macromolecular crystal during an X-ray diffraction experiment. The incident-beam parameters (X-ray flux density, photon energy and beam shape) and the crystal size, together with the absorption and attenuation coefficients obtained from knowledge of the total number of different atom types in the unit cell of the crystal, are used to calculate the absorbed dose. In the previous two versions of the program only the photoelectric cross-section was included in the calculations of the absorption coefficient. At the incident X-ray energies usually used in MX the Compton-scattering cross-section is very low and thus is negligible in dose calculations, but at higher energies (>20 keV) it increases and thus should be taken into account. For example, Compton-scattering events in a 100 µm thick crystal of chicken egg-white lysozyme at X-ray energies of 12.4 and 40 keV account for 5.4% and 67%, respectively, of the total number of interaction events (Fig. 1 ▶). In the new version (version 3) of *RADDOSE* reported here, the dose deposited by the Compton electron (owing to Compton scattering) is included in the calculation of the overall absorption coefficient to provide a better estimate of the absorbed dose at higher incident X-ray energies.

Following Arndt (1984 ▶), for a beam with incident intensity *I*_0_ (equal to the total number of photons incident on the crystal), the scattered intensity *I*_scatt_ is
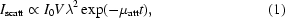
where *V* is the irradiated volume of the crystal, λ is the wavelength of the incident radiation and μ_att_ is the attenuation coefficient of the crystal with path length *t* in the beam. As the wavelength-dependence of radiation damage was then un­known, Arndt (1984 ▶) suggested that it would be an advantage to collect data at shorter wavelengths (higher energies). It was thought that at room temperature this might provide several possible benefits: *viz.* (i) reduced self-absorption of the diffracted X-rays by the sample, leading to lower *B* factors, (ii) improved data consistency across different samples, leading to more accurate estimation of isomorphous and anomalous differences, (ii) forward coning of the diffraction pattern, which allows longer crystal-to-detector distances to be used, hence improving the signal-to-noise ratio, (iv) improved phasing possibilities at the *K* and *L* edges of heavy elements and, most importantly, (v) improved sample lifetimes (Helliwell & Fourme, 1983 ▶; Helliwell *et al.*, 1993 ▶; Schiltz *et al.*, 1997 ▶). A recent study at 100 K of diffraction from 0.4 mm thick crystals at ultrahigh energies (55.6 keV) and at an energy routinely used in MX (12 keV) show a lower rate of radiation damage at ultrahigh energies in spite of longer data-collection times, owing to the lower deposited dose (Jakoncic *et al.*, 2006 ▶). It was demonstrated that sufficient signal can be obtained for phasing at ultrahigh energies. Shimizu *et al.* (2007 ▶) monitored radiation damage to chicken egg-white lysozyme crystals at nine different X-ray energies (6.5, 7.1, 8.3, 9.9, 12.4, 16.5, 20, 24.8 and 33 keV) and observed that the degradation of crystallographic statistics was independent of the incident energy but that the damage was proportional to the absorbed dose. It is important to note that the diffracted intensity per incident photon decreases as the incident energy is increased.

Complementing these efforts, there has also been considerable interest in using lower energy (longer wavelength) radiation in MX (Lehmann *et al.*, 1993 ▶; Stuhrmann *et al.*, 1995 ▶, 1997 ▶; Behrens *et al.*, 1998 ▶; Weiss *et al.*, 2005 ▶), since the diffracted intensity per incident photon is larger than at higher energies. Although the absorption is large at low energies for thick crystals, it is still small for thin crystals (Blundell & Johnson, 1976 ▶; Nave, 1995 ▶). Another possible advantage of lower energy radiation is the possible utilization of anomalous diffraction observed near the absorption edges of lighter elements such as sulfur (2.47 keV) and phosphorus (2.14 keV), which are both often present in macromolecules (Boesecke *et al.*, 2009 ▶). Other heavy atoms such as uranium (*M*_V_ = 3.5 Å) or a noble gas such as xenon (*L*_III_ = 2.6 Å) could also be used in phasing. Ultimately, an optimum X-ray diffraction experiment maximizes the scattered intensity of the diffraction spot (*I*_scatt_) while minimizing the absorbed dose (*D*). Here, we use *RADDOSE* to analyse the behaviour of the quantity *I*_scatt_/*D* (= *I*_DE_) in an attempt to understand the possible benefits of using high-energy and low-energy radiation.

This paper also aims to provide a guide for the practical use of the program *RADDOSE* under various circumstances by describing each of the necessary keywords. For additional details of the theory and methodology used by the program, the reader is referred to previous descriptions of *RADDOSE* and its usage (Murray *et al.*, 2004 ▶; Paithankar *et al.*, 2009 ▶).

## 2. Methods

### 2.1. Input

**Figure 2:**
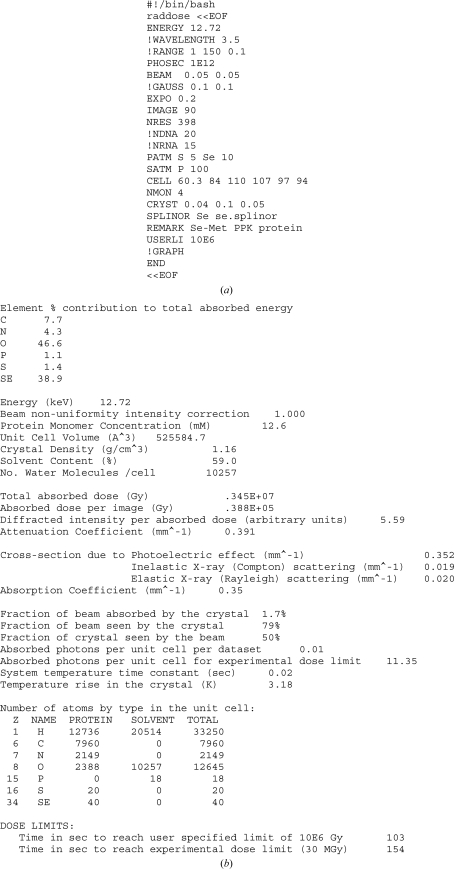
Example (*a*) input and (*b*) output files for the case study of data collections from PPK (see §[Sec sec3]3).

To compute the dose absorbed by the crystal, the program requires the crystal and beam characteristics for a particular experiment. The input file for running the program is given in Fig. 2 ▶(*a*): in the following description the keywords for the various parameters are shown in square brackets. The input requires the incident-beam energy *E* [EN] [*E *(keV) = 12.4/λ (in angstroms)] or wavelength λ [WAVE], along with the flux [PHOSEC] and beam size (*x* and *y* dimensions in millimetres; [BEAM]). The profile of the beam is taken to be a top-hat (boxcar or rectangular) shape, such that the beam shows uniform flux density over the entire profile. Alternatively, the full-width half-maxima (*x* and *y*) in millimetres of the beam can be provided by means of the keyword [GAUSS]. The time in seconds per exposure [EXPO] and the number of exposures [IMAGE] must also be supplied to the program. Appropriate methods to determine the flux of an X-­ray beam accurately for MX have recently been described in detail by Owen *et al.* (2009 ▶) and the necessary beam parameters, namely flux [PHOSEC] and shape [BEAM], can usually be obtained from the relevant beamline scientist at the synchrotron. In almost all cases the number of amino-acid residues [NRES] in the protein molecule is known to the crystallographer well before the experiment. In the case of nucleic acids, [NDNA] and [NRNA] can be used to supply information on the number of DNA and RNA nucleotides, respectively. The program assumes the following compositions for the various macromolecular entities: 5 C, 1.35 N, 1.5 O and 8 H atoms for an amino-acid residue, 9.75 C, 4 N, 6 O, 11.75 H and 1 P atom for a DNA nucleotide and 9.5 C, 3.75 N, 7 O, 11.25 H and 1 P atom for an RNA nucleotide. It is very beneficial to know of the presence of any heavy-metal atoms that are bound to the protein (for example, tantalum clusters), since they will greatly increase the atomic cross-section seen by the beam, *i.e.* the absorption coefficient. Information on the number of methionine residues or the number of DNA bases is also important for selenomethionine-derivatized proteins or DNA-containing complexes, respectively. The keyword [PATM] allows the number of non H, C, N or O atoms per protein molecule to be entered. Solvent molecules, either in the mother liquor or in the cryobuffers, play a vital part in the beam absorption and hence contribute to the absorbed dose, in particular if a crystal has been soaked in a heavy-atom solution. Solvent information can be supplied by using the keyword [SATM] (concentrations in millimoles). An important but often overlooked procedure is the back-soaking of the crystal in a heavy-atom-free solution to remove any non­specifically bound heavy atoms from the solvent channels. These nonspecifically bound heavy atoms contribute both to the absorption (*i.e.* they will increase the dose) and to the diffuse background, but do not contribute to the anomalous signal (Garman & Murray, 2003 ▶), and are thus undesirable.

*RADDOSE* also requires the cell dimensions [CELL] of the crystal under study, since it computes the volume not occupied by the macromolecule and fills it with solvent. The unit cell can be obtained by indexing a couple of exposures from the crystal collected with an attenuated beam. Using the program *MATTHEWS_COEF* (Matthews, 1968 ▶; Collaborative Com­putational Project, Number 4, 1994 ▶; Kantardjieff & Rupp, 2003 ▶), the probable number of monomers in the asymmetric unit can be determined and by multiplying this by the number of symmetry operators for that space group, the total number of protein molecules in one unit cell [NMON] can be com­puted. The parameter [CRYST] is used to supply the dimensions of the crystal (in millimetres: horizontal, vertical and thickness as seen by the X-ray beam).

The exact energies of the absorption edges of atoms can depend on their local environment and may differ widely from the values listed in databases. An experimental fluorescence scan can be collected and then normalized to the theoretical values below and above the absorption edge using the program *CHOOCH* (Evans & Pettifer, 2001 ▶); the resulting file can then be input into *RADDOSE* using the keyword [SPLINOR]. The photoelectric cross-sections used by the program to compute the absorption coefficients are derived from the McMaster databases implemented in the form of a subroutine (*mucal.f*; Badyopadhyay, 1995 ▶). The temperature rise induced by the X-ray beam in the sample is also calculated using the simple isothermal ‘lumped model’ (Kuzay *et al.*, 2001 ▶). The keyword [REMARK] is provided so that the user can add comments and identify the program output. In addition to the dose absorbed by the crystal, the program outputs the time (in seconds) taken to reach the experimental dose limit (30 MGy) and the ‘diffraction-dose efficiency’ (see §[Sec sec3]3). If desired, the keyword [USERLIMIT] can be used to output the time (in seconds) taken to reach a user-supplied dose limit (in grays) or the default value of 20 MGy (the ‘Henderson limit’). By using the keyword [RANGE], the dose for a range of energies under the specified conditions can be calculated and this can help in selecting the incident photon energy for a particular diffraction experiment. The option [GRAPH] gives the time taken to reach the experimental dose limit for a given range of energies in the form of a table, which can then be plotted for inspection.

### 2.2. Output

The most important parameters given by the output (Fig. 2 ▶ *b*) are (i) the absorbed dose for a given number of exposures of specified duration and (ii) the time taken to reach the experimental dose limit of 30 MGy (see §[Sec sec1]1) or to reach a user-specified dose. The attenuation coefficient (μ_att_; total cross-section) output by the program is composed of the photoelectric (μ_pe_), inelastic (*μ*_c_) and elastic (μ_r_) contributions, whereas the absorption coefficient (μ_abs_) includes only the photoelectric and inelastic contributions since for the elastic component there is no absorption.

The calculated temperature rise in the crystal, the number of absorbed photons per unit cell per data set, the fraction of the beam seen by the crystal and the number of atoms of each *Z* (atomic number) in the unit cell are also given in the output. These values, as well as the elemental contributions (% for each atom type) to the absorption coefficient, give more detailed information on the dose calculated from the supplied input parameters.

### 2.3. Incoherent (Compton) scattering correction

**Figure 3:**
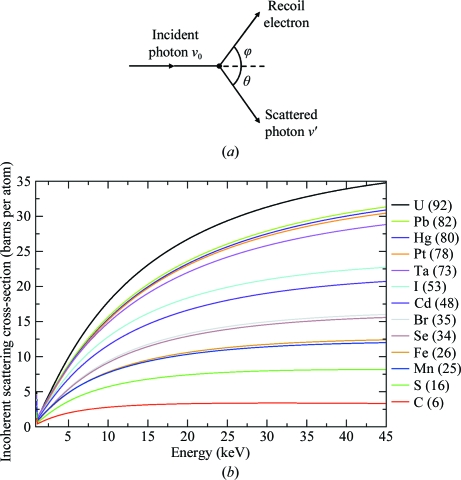
(*a*) The interaction of a photon with a free electron (figure adapted from §5.4 of Burcham, 1963 ▶). (*b*) Incoherent scattering cross-sections obtained from the XCOM tables (http://physics.nist.gov/PhysRefData/Xcom/html/xcom1.html) for elements of interest as a function of the incident photon energy.

Compton scattering is only one of the possible incoherent scattering processes that can occur, although the term is often loosely used to include plasmon, Raman and resonant Raman scattering. However, these additional processes are only significant compared with Compton scattering at much lower incident energies (<100 eV) than those used in MX. In our calculations, we therefore use the ‘incoherent’ scattering cross-sections tabulated by the National Institutes of Standards and Technology (NIST) but only consider the energy lost by the Compton electron in the crystal. As the incident photon energy increases (shorter wavelengths), instead of the incident photon interacting with the atom as a whole there is an increasing probability for the interaction to take place with individual electrons, provided a large enough momentum transfer takes place (from the photon to the electron). This collision of a photon with a quasi-free electron is known as the Compton effect. Whereas in Rayleigh scattering both the incident and scattered radiation are of the same wavelength, Compton-scattered radiation is of longer wavelength (than the incident beam) and its wavelength depends on the angle of scatter (Fig. 3 ▶ *a*). The probability of Compton scattering depends not only on the relevant atomic cross-section, but also on the details of the photon–electron interaction, *e.g.* the relation between the direction of polarization of the incident radiation and the direction of the spin momentum of the scattering electron. Given their small magnitude, the inclusion of these latter effects was considered to be beyond the scope of our work. A detailed discussion of incoherent scattering can be found in Burcham (1963 ▶) and *International Tables for Crystallography* (Wilson, 1995 ▶).

Fig. 3 ▶(*b*) shows the variation with energy of the incoherent scattering cross-section (McMaster *et al.*, 1969 ▶) per atom of various elements. The change in wavelength of a Compton-scattered photon is derived from conservation of energy and momentum, *i.e.* assuming an overall elastic event. For scattering of a free electron (Fig. 3 ▶ *a*) by an incident photon of wavelength λ_0_ and frequency ν_0_, the change in the wavelength of the Compton-scattered photon, Δλ, can be derived as
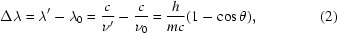
where λ′ (ν′) is the wavelength (frequency) of the scattered photon, *m* corresponds to the electron rest energy of 511 keV/*c* ^2^, *c* is the velocity of light and θ is the angle between the incident-beam direction and the Compton-scattered radiation (as shown in Fig. 3 ▶ *a*). The energy of the scattered photon is given by
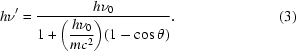
The recoil electron, initially at rest, acquires a kinetic energy given by
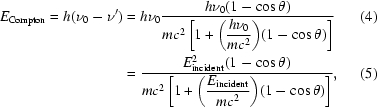
where *E*_incident_ is the energy of the incident radiation of frequency ν_0_ and *h* is Planck’s constant. Depending on the scattering angle of the scattered photon, the energy of the recoil Compton electron varies between zero for θ = 0 to a maximum value at θ = π. (5) can be analytically integrated over θ from θ = 0 to θ = π and divided by π to give the average Compton electron energy,
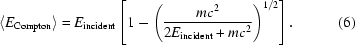

            

### 2.4. Dose calculation

The intensity of the incident beam absorbed by the crystal, *I*_A_, is given by
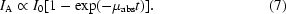
The total energy absorbed by the crystal, *E*_T_, is proportional to the incident-beam intensity and is given by
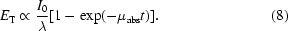
The total dose, *D* [*D* = (absorbed energy/mass) in J kg^−1^] is then computed from both the photoelectric (μ_pe_) and the Compton (μ_c_) contributions, 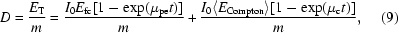

               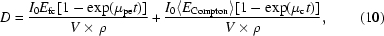
where *m* = V × ρ, *m*, *V* and *ρ* are the irradiated mass and volume and the density of the crystal, respectively, and, as before, *t* is the thickness of the sample as seen by the beam. *E*_fc_ is the energy deposited in the crystal by a photoelectric absorption event after taking into account the probability of X-ray fluorescent escape as included in *RADDOSE* v.2 and reported previously (Paithankar *et al.*, 2009 ▶). For computational convenience (10) is not further simplified in the program.

## 3. Discussion

**Figure 4:**
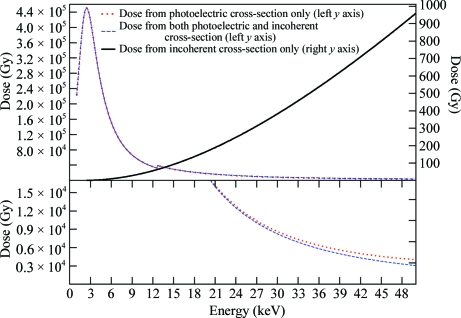
Calculated dose absorbed by a crystal of the protein PPK with and without the inclusion of Compton scattering. The calculations were performed assuming a crystal size of 0.04 × 0.1 × 0.05 mm, a top-hat-shaped beam of 0.05 × 0.05 mm with a photon flux of 10^12^ photons s^−1^ and an exposure time of 0.2 s per image. The protein consists of 398 residues, crystallizes in space group *P*1 with four monomers in the unit cell and contains three cysteines and 12 methionines (excluding the N-­terminal methionine residue) with approximately 58% solvent content. The lower panel is an enlargement of the 10–200 kGy dose region of the upper panel, highlighting the small difference between the absorbed dose with and without the inclusion of Compton scattering at higher incident energies.

As an aid to investigating the advantages and disadvantages of collecting data at various energies, the dose absorbed by the protein phosphofructokinase (PPK; UniProtKB accession code Q9WZP7) from *Thermotoga maritima* (E. Rudiňo-Piňera, unpublished results) at various energies including or excluding Compton scattering [*i.e.* with and without the second term in (10)] is plotted in Fig. 4 ▶. It can be seen that photoelectric absorption is the dominant process at low energies, but as the incident energy increases the probability of Compton scattering increases (with a peak around 80 keV; data not shown) while photoelectric absorption decreases. Overall, the rate of increase in Compton scattering with increasing incident energy is much lower than the rate of decline in the probability of photoelectric absorption, which is higher in magnitude. Thus, in spite of the increase in the Compton cross-section with energy, the total dose (photoelectric plus Compton con­tribution) decreases as the incident energy increases, with a shallow minimum at around 80 keV, after which the total dose slowly increases again.

An optimum experimental protocol involves finding the energy of the incident beam which maximizes the intensity of the diffracted photons with the minimum amount of energy deposited per kilogram (*i.e.* to the dose) in the sample, the so-called ‘diffraction-dose efficiency’ *I*_DE_ (Murray *et al.*, 2004 ▶).

**Figure 5:**
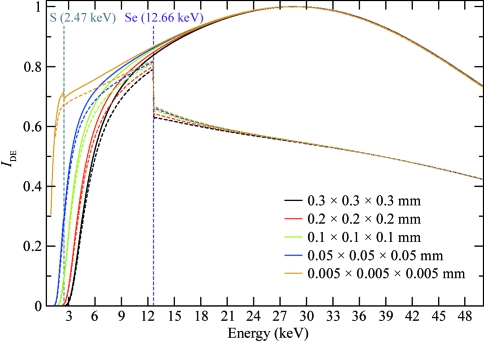
*I*_DE_ for native (solid lines) and SeMet-derivative (broken lines; ten Se atoms measured by microPIXE; Garman & Grime, 2005 ▶) crystals of the protein PPK of various sizes. For a given crystal size, the value of *I*_DE_ was normalized to unity at the peak value for the native form of the crystal. All calculations shown here were performed assuming a top-hat-shaped beam of size equal to that of the crystal with a photon flux of 10^12^ photons s^−1^ and an exposure time of 0.2 s per image and took into account both X-ray fluorescent escape and Compton scattering. It is clear that diffraction-dose efficiency (*I*_DE_) reaches a peak for crystals of all sizes at photon energies of 24–34 keV.

Using the program *RADDOSE*, we here investigate the behaviour of *I*_DE_ to elucidate the search for optimum MX experimental conditions. Re-examination of this parameter illustrates the importance of the volume term in the equations for diffraction-dose efficiency (*I*_DE_) which was omitted in previous treatments [see equation (7) of Murray *et al.* (2004 ▶) and equation (4) of Paithankar *et al.* (2009 ▶) in which the *V* ^2^ terms are missing]. Dividing equation (1) for the diffracted intensity by equation (10) for the dose leads to 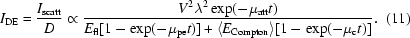
In the above equation, ρ is included in the proportionality sign. A plot of diffraction-dose efficiency (*I*_DE_; normalized to unity at the peak value of *I*_DE_ for each crystal size) for PPK crystals of various sizes taking into account both X-ray fluorescent escape and Compton scattering is shown in Fig. 5 ▶. It is clear that *I*_DE_ reaches a peak for native crystals of all sizes at incident photon energies of between 24 and 34 keV. At lower energies *I*_DE_ is significantly lower for all crystal sizes but shows reduced sensitivity for thinner crystals. Notably, the diffraction-dose efficiency for thicker crystals at low energies (5 keV) is only one quarter of the *I*_DE_ value at 12.4 keV, but does not drop nearly as steeply for thinner crystals. In the case of the selenomethionine derivative of PPK, the effect of the ten Se atoms per PPK molecule can clearly be seen in Fig. 5 ▶, since *I*_DE_ falls suddenly at the absorption edge, where the selenium photoelectric cross-section substantially increases. It should be pointed out that the behaviour of *I*_DE_ is similar to the behaviour of the time it would take to reach the experimental dose limit, *i.e.* a higher value of *I*_DE_ implies that a longer crystal lifetime is predicted under the same conditions.

Nave & Hill (2005 ▶) and Cowan & Nave (2008 ▶) examined the ‘ratio of dose to scattered intensity of a diffraction spot’ (which is the reciprocal of the diffraction-dose efficiency) for very small samples (<4 µm) by Monte Carlo simulations. The realization that a significant proportion of the photoelectrons could escape the crystal (in spite of the photoelectron having a nonlinear path) introduces the possibility of taking advantage of the reduced energy deposition in very small crystals and this effect was included in their calculations. In their simulations the likelihood of X-ray fluorescent escape (non-negligible when collecting data above the absorption edge in the pre­sence of heavy atoms) was not taken into account and this can also have a significant effect on reducing the dose (Paithankar *et al.*, 2009 ▶). They concluded that there is a worthwhile advantage in collecting data at higher energies (20–30 keV) for small crystals (1–20 µm) since the photoelectron would then have a high probability of escaping from the crystal. Our calculations using *RADDOSE* show that an increase in energy beyond 30 keV does not improve the value of *I*_DE_, which is similar to predictions made by Cowan & Nave (2008 ▶). As mentioned previously, the photoelectric cross-section decreases with increasing energy at a faster rate than the Compton cross-section increases, leading to a reduction in the dose at high (>25 keV) energies. Thus, the lower values of *I*_DE_ (for native crystals) at energies >30 keV can be attributed (at least partially) to the decrease in Rayleigh cross-section with an increase in energy (Fig. 1 ▶). The fluorescent escape correction becomes more important at high incident energies and for thin crystals, especially for those with heavy anomalous scatterers and which previous (theoretical) studies have not taken into account.

**Figure 6:**
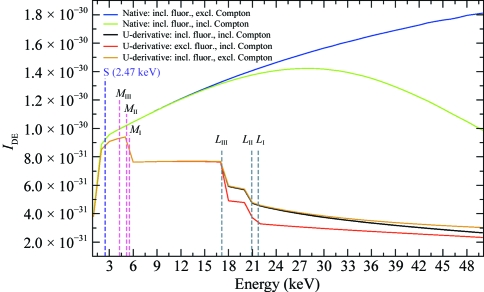
*I*_DE_ for native and uranium-derivative crystals of human phosphatase-binding protein (HPBP) with (hypothetical) dimensions of 0.005 × 0.005 × 0.005 mm. All calculations shown here were performed assuming a top-hat-shaped beam of size equal to that of the crystal with a photon flux of 10^12^ photons s^−1^ and an exposure time of 1 s per image.

To illustrate the significant effect of heavy atoms on the value of *I*_DE_, calculations for data collections on crystals of human phosphatase-binding protein (HPBP) are used here as an example (Morales *et al.*, 2006 ▶). From Fig. 6 ▶, it is clear that crystals with heavy anomalous scatterers show lower *I*_DE_ at energies above the respective absorption edge. The benefits of energy loss from the crystal through X-ray fluorescent escape (from thin crystals) are outweighed by the enormous increase in the photoelectric cross-section above the edge energy. This shows that while it may be beneficial to conduct native data collection at 24–34 keV, experiments in the presence of anomalous scatterers at very high energies may be detrimental to the sample and lower the *I*_DE_. Thus, the use of long-wavelength radiation (<4 keV) showing higher values of *I*_DE_ is more promising for thinner crystals than for thicker crystals (Figs. 5 ▶ and 6 ▶). This is consistent with the predictions of Nave (1995 ▶) on the use of long-wavelength radiation. The predictions shown in Fig. 5 ▶ reinforce the possible benefits of the development of long-wavelength beamlines which may be advantageous both in terms of the optimum utilization of microcrystals and the capacity to exploit both endogenous phasing (from atoms such as sulfur) and that from conventional heavy-atom soaks.

## 4. Conclusions

The program *RADDOSE* now encompasses all the physical events that occur in a crystal with the exception of photoelectron escape (which is only significant in the case of very small crystals and high-energy incident X-ray radiation). *RADDOSE* calculations provide reasonable estimates for the dose absorbed by a crystal in a swift manner. The program has now been seamlessly integrated into the *EDNA* project (Leslie *et al.*, 2002 ▶; Incardona *et al.*, 2009 ▶) for user-friendly operation at many beamlines and also can be used in con­junction with *BEST* (Bourenkov & Popov, 2010 ▶) to optimize data-collection strategies. It should be noted that the program *RADDOSE* assumes that the crystal is stationary during irradiation. For a crystal that is smaller than (or equal to) the size of the beam this gives no error. However, when the crystal is larger than the beam size this assumption causes the dose to be overestimated, since as the crystal is rotated new non-irradiated parts of the crystal enter the beam. In order to take this into account, both the accurate dimensions of the crystal and the orientation of these dimensions relative to the physical rotation axis must be known. Tools for providing this information are currently being developed at modern synchrotron beamlines. Eventually, it will thus be possible to arrive at a better estimate of the absorbed dose for crystals that are larger than the beam, knowledge of which will allow improved data-collection strategies.

The results presented here indicate that high-energy X-ray beams might allow a reduction in the rate of radiation damage for the same diffraction intensities, but may not be very useful for thin samples containing heavy atoms. Complementary methods using long-wavelength radiation with specialized instrumentation, detectors and software (to correct for absorption errors) may be preferred in such cases in order to exploit the increased scattering power and phasing possibilities in a routine manner.

The program *RADDOSE* (version 3) can be obtained by emailing the authors at elspeth.garman@bioch.ox.ac.uk.
